# Osteopenia Is Associated with Shorter Survival in Patients with Intrahepatic Cholangiocarcinoma

**DOI:** 10.3390/curroncol30020144

**Published:** 2023-02-02

**Authors:** Atsushi Miki, Yasunaru Sakuma, Jun Watanabe, Kazuhiro Endo, Hideki Sasanuma, Takumi Teratani, Alan Kawarai Lefor, Joji Kitayama, Naohiro Sata

**Affiliations:** Department of Surgery, Division of Gastroenterological, General and Transplant Surgery, Jichi Medical University, 3311-1 Yakushiji, Shimotsuke 329-0498, Tochigi, Japan

**Keywords:** biomarker, sarcopenia, osteopenia, myosteatosis

## Abstract

Background: The prognostic importance of osteopenia in patients with intrahepatic cholangiocarcinoma (ICC) undergoing hepatectomy is unclear. The aim of this study was to evaluate the impact of osteopenia on survival in patients with ICC. Methods: A total of 71 patients who underwent hepatectomy at Jichi Medical University between July 2008 and June 2022 were included in this study. Non-contrast computed tomography scan images at the eleventh thoracic vertebra were used to assess bone mineral density. The cutoff value was calculated using a threshold value of 160 Hounsfield units. Overall survival curves were made using the Kaplan–Meier method and the log-rank test was used to evaluate survival. The hazard ratio (HR) and 95% confidence interval (CI) for overall survival were calculated using Cox’s proportional hazard model. Results: In multivariable analysis, osteopenia (HR 3.66, 95%CI 1.16–14.1, *p* = 0.0258) and the platelet–lymphocyte ratio (HR 6.26, 95%CI 2.27–15.9, *p* = 0.0008) were significant independent factors associated with overall survival. There were no significant independent prognostic factors for recurrence-free survival. Conclusions: Preoperative osteopenia is significantly associated with postoperative survival in patients with ICC undergoing hepatectomy.

## 1. Introduction

Intrahepatic cholangiocarcinoma (ICC) is the second most common primary liver malignancy [1]. ICC, a rare primary malignant tumor of the liver, is thought to originate from cholangioles or canals of Hering [2]. Complete surgical resection is the only method to achieve long-term survival, with an estimated 5-year survival rate of 30% to 35%, although many patients develop recurrence postoperatively [3]. Although combined systemic chemotherapy has been demonstrated to improve survival, patients with this malignancy still have a poor prognosis, even when resected and treated with chemotherapy [4,5]. Traditionally, the classification scheme in the American Joint Committee on Cancer (AJCC) staging manual has been used to stratify patients with regard to prognosis. AJCC staging of ICC includes factors such as the number of tumors and the presence/absence of vascular invasion, as well as involvement of adjacent structures [6].

Cancer cachexia is a destructive syndrome characterized by atrophy of deep skeletal muscle, with or without changes in adiposity, found in 63% of patients at diagnosis and in 80% of patients as the disease progresses [7,8]. Cachexia decreases an individual’s quality of life and tolerance to cancer treatment and is associated with poor clinical outcomes and survival [9]. There are multiple definitions of cachexia, but most consider it to be a loss of weight as well as a loss of quantity and quality of skeletal muscle [9]. Sarcopenia refers to age-related loss of muscle strength, muscle mass, and functionality, and is associated with osteoporosis, frailty, and cachexia [10]. Several studies have investigated the relationship between muscle biology and bone density in normal aging and various neuromuscular diseases [11,12,13]. Bone and muscle changes are thought to occur continuously due to inflammation, general undernutrition, malnutrition, and reduced physical activity [13].

Osteopenia is defined as a decrease in protein and mineral content of bone tissue but is not as severe as osteoporosis. Recently, osteopenia was reported to be associated with poor prognosis in patients undergoing liver transplantation for hepatocellular carcinoma, by Sharma et al. [14]. Bone mineral density (BMD) was analyzed by assessing the average pixel density of trabecular bone in the thoracic vertebrae on preoperative computed tomography (CT) scans. Dual-energy X-ray absorptiometry is the standard for assessing BMD, but CT scan-based attenuation values are increasingly being used to characterize BMD because they are widely used as part of preoperative staging of these patients [15]. Recently, preoperative osteosarcopenia was reported to be a predictor of adverse prognosis for patients undergoing hepatic resection for ICC [16]. Osteosarcopenia is a condition in which osteopenia and sarcopenia occur simultaneously and is different from osteopenia alone or sarcopenia alone. However, there is little data regarding the prognostic value of osteopenia in patients with ICC undergoing hepatectomy. The aim of this study was to evaluate the clinical impact of osteopenia in patients with ICC. In the present study, osteopenia was a risk factor for five-year survival in multivariate analysis. Osteopenia, but not osteosarcopia or sarcopenia, was an independent prognostic factor.

## 2. Materials and Methods

### 2.1. Patients and Treatment

A total of 71 patients who underwent hepatectomy at Jichi Medical University Hospital between July 2008 and June 2022 were included in this study. There were no patients who died within 1 month postoperatively. The protocol for this study was approved by the Institutional Review Board and complies with the provisions of the Declaration of Helsinki (A22-046). Blood samples were collected within 2 weeks before surgery. An R0 resection was defined as complete resection of the tumor visible grossly by the surgeon with microscopically negative margins on histopathologic examination, while an R1 resection was defined as complete resection of the tumor visible grossly and microscopically positive margins on histopathologic examination. An R2 resection was defined as resection of the tumor visible grossly but leaving macroscopic residual disease because the tumor was present beyond the resection line. Surgical procedures included right hemi-hepatectomy in 9 patients, left hemi-hepatectomy in 25 patients, segmentectomy in 8 patients, sub-segmentectomy in 10 patients, limited resection in 7 patients, central bi-segmentectomy in 2 patients, R2 resection in 8 patients, and right tri-segmentectomy in 2 patients. Adjuvant chemotherapy was administered with gemcitabine, S-1, or both, and the duration of adjuvant chemotherapy was at least 6 months. Overall survival (OS) was delimited from the date of surgery to the date of death or last contact with the patient (censored). Staging of ICC patients followed the American Joint Committee on Cancer 8th edition staging system.

The techniques for hepatic resection were classified according to the Brisbane Nomenclature of the International Society for Hepato-Pancreatico-Biliary Research [17]. The procedure was classified as a hemi-hepatectomy, an extended hemi-hepatectomy (hepatectomy plus removal of additional contiguous segments), a sectionectomy (resection of two Couinaud subsegments), or a segmentectomy (resection of one Couinaud subsegment). All other non-anatomic procedures were classified as limited resections [18].

The prognostic nutrition index was measured using the formula: (10 × Albumin level) + (0.005 × lymphocyte level), and the cutoff value was set as 40, as previously reported [19]. The neutrophil–lymphocyte ratio was calculated using the formula: neutrophil level/lymphocyte level. The cutoff value was set using the receiver operating curve method as 1.9 (AUC = 0.56). The platelet–lymphocyte ratio (PLR) was calculated using the formula: platelet level/lymphocyte level, and the cutoff value was set using the receiver operating curve method as 0.16 (AUC = 0.53).

### 2.2. Definition of Osteopenia

BMD was assessed by analysis of CT scan images obtained within 3 months before surgery. Non-contrast CT scan images at the level of the 11th thoracic vertebra were used. BMD was measured by calculating the average pixel density within a circle at the center of the vertebral body to measure trabecular bone, as described in a previous study [20]. The cutoff value was calculated using a threshold value of 160 Hounsfield units (HU) for CT scans, as determined in a previous study [14].

### 2.3. Definition of Sarcopenia and Myosteatosis

Preoperative CT scan images at the third lumbar spine (L3) level were used to measure the psoas muscle mass index (PMI), which is used to quantify skeletal muscle mass. PMI was calculated by dividing the cross-sectional area of the psoas major muscle by the square of its height (cm^2^/m^2^) [20]. The cutoff values for PMI for gender differences were set as previously reported: 6.36 for men and 3.92 for women [21]. Intramuscular adipose tissue content was calculated as the region of interest of the multifidus muscle (HU) divided by the region of interest of subcutaneous fat (HU) using images from the preoperative CT scan. The myosteatosis cutoff value was set as −0.0215 intramuscular adipose tissue content (area under the curve = 0.55) using the receiver operating curve method for overall survival.

### 2.4. Statistical Analysis

Continuous variables are presented as mean ± standard deviation and categorical variables are expressed as numbers. All categorical data were analyzed by Pearson’s chi-squared test. Normally distributed values were analyzed by Student’s *t*-test. Non-normally distributed values were analyzed by the Mann–Whitney U-test. The cutoff values for serum cancer embryonic antigen and carbohydrate antigen 19-9 were 4.5 mg/dl and 37 IU/mL, respectively, based on institutional data. The overall survival curves were constructed using the Kaplan–Meier method. A log-rank test was performed for survival using the Kaplan–Meier method. The hazard ratio (HR) and 95% confidence interval (CI) for overall survival were calculated using Cox’s proportional hazard model. All statistical analyses were performed using JMP version 16.0 (SAS Institute Inc., Cary, NC, USA). The significance threshold was set at *p* < 0.05.

## 3. Results

### 3.1. Patient Characteristics

The mean age of patients in the present study was 68.3 ± 8.6 years. This study included 46 (65%) males and 25 (35%) females. There were significant differences between normal BMD and osteopenia patients with regard to age (*p* = 0.0095), gender differences (*p* = 0.0211), and tumor pathological differentiation (*p* = 0.0327) (Table 1). There were no significant differences between normal BMD and osteopenia patients regarding the incidence of myosteatosis (*p* = 0.3829), sarcopenia (*p* = 0.6982), American Society of Anesthesiologists Score (*p* = 0.8000), neutrophil–lymphocyte ratio (*p* = 0.7498), prognostic nutrition index (*p* = 0.4096), intraoperative blood loss (*p* = 0.1601), carcinoembryonic antigen level (*p* = 0.2899), carbohydrate antigen 19-9 level (*p* = 0.1880), rate of R0 resection (*p* = 0.2464), stage (*p* = 0.6956), T stage (*p* = 0.8285), and nodal status (*p* = 0.3980).

### 3.2. Survival

**Figure 1:**
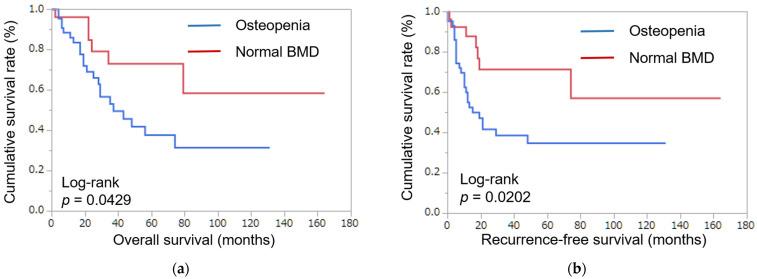
Patient survival. (**a**) Postoperative overall survival, classified by bone mineral density. (**b**) Postoperative recurrence-free survival, classified by bone mineral density. Normal BMD was better than osteopenia in five-year overall survival and recurrence-free survival in patients with ICC.

Using Kaplan–Meyer analysis, the 5-year overall survival rate in patients with normal BMD was 73.1% and the 5-year overall survival rate in patients with osteopenia was 37.8% (*p* = 0.0429) (Figure 1a). The 5-year recurrence-free survival rate in patients with normal BMD was 71.4% and the 5-year recurrence-free survival rate in patients with osteopenia was 34.9% (*p* = 0.0202) (Figure 1b). In Kaplan–Meyer analysis, normal BMD was better than osteopenia in five-year overall survival and recurrence-free survival.

### 3.3. Univariable and Multivariable Analysis for Overall Survival

In univariable analysis, osteopenia (HR 2.46, 95%CI 1.06–6.69, *p* = 0.0492), sarcopenia (HR 3.08, 95%CI 0.13–0.99, *p* = 0.0492), neutrophil–lymphocyte ratio (HR 6.26, 95%CI 2.27–15.9, *p* = 0.0008), PLR (HR 12.78, 95%CI 3.76–39.4, *p* = 0.0002), and lymph node status (HR 3.00, 95%CI 1.23–6.95, *p* = 0.0169) were factors significantly associated with overall survival (Table 2). Multivariate regression analysis among the variables showed statistically significant differences following univariate analysis for overall survival. In multivariable analysis, osteopenia (HR 3.66, 95%CI 1.16–14.1, *p* = 0.0258) and PLR (HR 6.26, 95%CI 2.27–15.9, *p* = 0.0008) were independent prognostic factors significantly associated with overall survival.

### 3.4. Univariable and Multivariable Analysis for Recurrence-Free Survival

In univariable analysis, gender (HR 0.49, 95%CI 0.25–0.89, *p* = 0.0464), osteopenia (HR 2.57, 95% CI 1.28–6.44, *p* = 0.0168), neutrophil–lymphocyte ratio (HR 5.34, 95%CI 2.12–12.4, *p* = 0.0008), and PLR (HR 12.77, 95%CI 3.58–43.7, *p* = 0.0003) were prognostic factors significantly associated with recurrence-free survival (Table 3). Multivariate regression analysis between variables showed no statistically significant differences following univariate analysis for overall survival. In multivariable analysis, no independent prognostic factors were significantly associated with recurrence-free survival.

## 4. Discussion

Osteopenia and PLR are independent prognostic factors significantly associated with overall survival. A previous report showed that osteosarcopenia is an independent prognostic factor, however, to the best of our knowledge, the present study is the first to evaluate the prognostic significance of osteopenia in patients undergoing hepatic resection for ICC. This study demonstrated that osteopenia is a stronger prognostic factor than sarcopenia or osteosarcopia.

It is unclear why osteopenic patients with cancer have a poor prognosis. Osteopenia and sarcopenia are features of the aging process that result in falls, fractures, and frailty in the elderly [22]. The development of frailty is a consequence of normal aging. Frailty is clinically used to classify people with cancer who are at risk for poor outcomes, and after therapy for cancer, to identify survivors at risk for early morbidity and mortality [23]. Impairment of body composition, such as loss of muscle mass (sarcopenia) as well as muscle quality (myosteatosis), have been shown to affect perioperative outcomes for various diseases [24,25,26,27].

The influence of a low BMD on prognostic outcome may be related to immune function. Recent studies have investigated the potential interactions between bone and the immune system, known as ‘‘osteoimmunology’’ [28]. The pathophysiology for the progression of sarcopenia or osteopenia in patients with advanced cancers is unclear. One possibility is that NF-κB is a key molecule associated with sarcopenia [29]. Receptor activator of the NF-κB (RANK) ligand activates, while osteoprotegerin inhibits, osteoclastogenesis, leading to osteopenia [30]. RANK is also expressed in skeletal muscle, and activation of the NF-κB pathway primarily inhibits muscle differentiation, leading to loss or impairment of skeletal muscle function, which cause sarcopenia [31,32]. NF-κB promotes migration and invasion by upregulating Snail expression in cholangiocarcinoma cells, which in turn suppresses E-cadherin [33]. Since cancer and cachexia are inflammatory responses, it is reasonable to assume that the diverse clinical manifestations of debilitation and osteopenia are related via inflammatory cytokines from a variety of origins.

There was no significant association between osteopenia and recurrence-free survival. Sharma et al. also showed that bone loss was independently associated with mortality after liver transplantation in patients with hepatocellular carcinoma but not with recurrence, which is the same result found in the present study [14]. Although hepatocellular carcinoma and ICC are different diseases, it suggests the possibility that something similar is occurring in patients with osteopenia.

PLR was found to be a significant independent prognostic factor in the present study. The preoperative systemic inflammation response (neutrophil–lymphocyte ratio and PLR) has been shown to be independently associated with cancer-specific survival in patients undergoing curative resection of several types of solid tumors [34,35,36]. Chen et al. have recently demonstrated that the PLR was an independent predictor of recurrence and poor overall survival in patients with ICC [37]. Tumor progression and metastasis formation is the result of dynamic interactions between tumor cells themselves and components of the tumor inflammatory environment [38]. High PLR values may reflect relatively depleted lymphocytes, which impairs the host immune response to malignancy [37]. Both immune and nutritional status play crucial roles in cancer progression and prognosis [39,40].

The impact of nutritional therapy on prognosis after liver transplantation was reported in [41]. In addition, several studies have already reported that preoperative rehabilitation is effective in reducing postoperative complications in patients with lung cancer, colorectal cancer, esophageal cancer, and other cancers [42,43,44]. Such supportive care, focusing on nutrition and rehabilitation, would be applicable to patients with ICC, but the effectiveness of supportive care must be evaluated in a prospective trial.

Age was associated with osteopenia, but multivariate analysis revealed that it was not associated with survival as strongly as osteopenia. Most deaths were cancer-related deaths, which were not considered to be related to age.

Patients with cholestatic liver disease are particularly prone to osteoporosis because biliary stasis interferes with vitamin D metabolism. However, there were few patients who manifested cholestatic liver disease in the present study. Further study is necessary to elucidate the role of the liver in osteopenia.

This study has several acknowledged limitations. First, this study was conducted retrospectively at a single institution with a small sample size. However, confounding factors were evaluated using descriptive statistics and univariate analysis and adjusted for whenever possible. In addition, preoperative CT and blood tests were routinely performed within three months prior to surgery, with limited risk of observational bias. The results presented here should be prospectively validated in a multicenter study with a larger population. Second, the cutoff values for the diagnosis of osteopenia used in the present study were based on previous reports [14,45], but further studies are needed to evaluate whether these values are appropriate.

## 5. Conclusions

Preoperative osteopenia is associated with decreased postoperative survival in patients with ICC undergoing hepatectomy. Preoperative assessment of osteopenia, along with tumor-specific prognostic factors, is important for risk stratification and decision-making in patients with ICC. Adequate nutrition and physical rehabilitation for ICC may improve bone mineral loss and increase motor function and may contribute to an improved postoperative prognosis.

## Figures and Tables

**Table 1:** Clinicopathological characteristics of patients.

Variables	Normal BMD (*n* = 27)	Osteopenia (*n* = 44)	*p*-Value
Age (y), mean ± SD	64.9 ± 9.7	70.3 ± 7.2	0.0095
Gender, Female vs. Male	5 vs. 22	20 vs. 24	0.0211
Myosteatosis, yes vs. no	21 vs. 6	30 vs. 14	0.3829
Sarcopenia, yes vs. no	23 vs. 4	38 vs. 5	0.6982
ASA Score, 1, 2 vs. 3	23 vs. 3	38 vs. 6	0.8000
Neutrophil–lymphocyte ratio, mean ± SD	4.3 ± 5.7	3.9 ± 3.8	0.7498
Platelet–lymphocyte ratio, mean ± SD	0.18 ± 0.12	0.17 ± 0.13	0.9355
Prognostic Nutrition Index, mean ± SD	37.7 ± 6.6	38.9 ± 4.1	0.4096
Intraoperative blood loss, mean ± SD	948 ± 940	1437 ± 1454	0.1601
CEA (mg/dl), mean ± SD	3.8 ± 3.4	5.3 ± 6.2	0.2899
CA19-9 (IU/mL), mean ± SD	5380 ± 23,780	570 ± 1130	0.1880
R0 resection, yes vs. no	22 vs. 5	40 vs. 4	0.2464
Stage, I, II vs. III, IV	19 vs. 8	29 vs. 15	0.6956
Differentiation, well, moderate vs. poorly	18 vs. 4	38 vs. 1	0.0327
T stage, I, II vs. III, IV	20 vs. 2	37 vs. 3	0.8285
N stage, positive vs. negative	3 vs. 19	9 vs. 31	0.3980

SD, standard deviation; ASA, American Society of Anesthesiologists; CEA, carcinoembryonic antigen; CA, carbohydrate antigen; T, tumor; N, node.

**Table 2:** Univariable and multivariable analysis for overall survival.

Variables	Univariable			Multivariable		
	HR	95% CI	*p*-Value	HR	95% CI	*p*-Value
Age (y), ≥70	1.85	0.87–4.07	0.1083			
Gender, male	0.69	0.32–1.52	0.3431			
Osteopenia, yes	2.46	1.06–6.69	0.0357	3.66	1.16–14.1	0.0258
Myosteatosis, yes	1.73	0.71–5.14	0.2423			
Sarcopenia, yes	3.08	0.13–0.99	0.0492	2.89	0.62–10.4	0.1593
ASA Score, 1 or 2	0.82	0.28–3.48	0.7535			
Neutrophil–lymphocyte ratio, ≥1.9	6.26	2.27–15.9	0.0008	1.40	0.07–7.93	0.7650
Platelet–lymphocyte ratio, ≥0.16	12.78	3.76–39.4	0.0002	17.23	1.80–401	0.0126
Prognostic Nutrition Index, ≥40	0.51	0.19–1.25	0.1420			
Intraoperative blood loss, ≥760 mL	2.05	0.91–5.01	0.0816			
CEA (mg/dl), ≥4.5	2.02	0.92–4.39	0.0803			
CA19-9 (IU/mL), ≥37	2.16	0.95–5.52	0.0658			
R0 resection, no	1.15	0.27–3.30	0.8179			
Stage, I or II	2.01	0.92–4.25	0.0773			
Differentiation, well or moderate	0.84	0.05–4.01	0.8597			
T stage, 1 or 2	0.37	0.02–1.79	0.2635			
N stage, positive	3.00	1.23–6.95	0.0169	1.82	0.60–5.24	0.2799

ASA, American society of anesthesiologist; CEA, carcinoembryonic antigen; CA, carbohydrate antigen; T, tumor; N, node.

**Table 3:** Univariable and multivariable analysis for recurrence-free survival.

Variables	Univariable			Multivariable		
	HR	95% CI	*p*-Value	HR	95% CI	*p*-Value
Age (y) ≥70	1.61	0.82–3.28	0.1735			
Gender, male	0.49	0.25–0.89	0.0464	0.50	0.20–1.21	0.1230
Osteopenia, yes	2.57	1.28–6.44	0.0168	1.67	0.62–5.03	0.3158
Myosteatosis, yes	1.50	0.69–3.75	0.3255			
Sarcopenia, yes	2.05	0.76–4.71	0.1447			
ASA Score, 1 or 2	1.12	0.44–3.78	0.8323			
Neutrophil–lymphocyte ratio, ≥1.9	5.34	2.12–12.4	0.0008	3.17	0.67–11.4	0.1311
Platelet–lymphocyte ratio, ≥0.16	12.77	3.58–43.7	0.0003	4.11	0.85–21.0	0.0795
Prognostic Nutrition Index, ≥40	0.71	0.31–1.55	0.3918			
Intraoperative blood loss, ≥760 mL	2.05	0.98–4.53	0.0566			
CEA (mg/dl), ≥4.5	1.57	0.75–3.20	0.2214			
CA19-9 (IU/mL), ≥37	2.10	0.98–5.02	0.0561			
R0 resection, no	2.14	0.95–4.30	0.0637			
Stage, I or II	1.71	0.84–3.41	0.1366			
Differentiation, well or moderate	0.46	0.03–2.17	0.3923			
T stage, 1 or 2	1.41	0.42–8.78	0.6206			
N stage, positive	2.14	0.95–4.53	0.0637			

ASA, American society of anesthesiologist, CEA, carcinoembryonic antigen; CA, carbohydrate antigen; T, tumor; N, node.

## Data Availability

Our database contains highly sensitive data which may provide insight into clinical and personal information about our patients and lead to identification of these patients. Therefore, according to organizational restrictions and regulations, these data cannot be made publicly available. However, the datasets used and/or analyzed during the current study are available from the corresponding author upon reasonable request.
